# Combining S-cone and luminance signals adversely affects discrimination of objects within backgrounds

**DOI:** 10.1038/srep20504

**Published:** 2016-02-09

**Authors:** Ben J. Jennings, Konstantinos Tsattalios, Ramakrishna Chakravarthi, Jasna Martinovic

**Affiliations:** 1 McGill Vision Research, Department of Ophthalmology, Montreal General Hospital, Montreal, Quebec, Canada; 2 School of Psychology, University of Aberdeen, UK

## Abstract

The visual system processes objects embedded in complex scenes that vary in both luminance and colour. In such scenes, colour contributes to the segmentation of objects from backgrounds, but does it also affect perceptual organisation of object contours which are already defined by luminance signals, or are these processes unaffected by colour’s presence? We investigated if luminance and chromatic signals comparably sustain processing of objects embedded in backgrounds, by varying contrast along the luminance dimension and along the two cone-opponent colour directions. In the first experiment thresholds for object/non-object discrimination of Gaborised shapes were obtained in the presence and absence of background clutter. Contrast of the component Gabors was modulated along single colour/luminance dimensions or co-modulated along multiple dimensions simultaneously. Background clutter elevated discrimination thresholds only for combined S-(L + M) and L + M signals. The second experiment replicated and extended this finding by demonstrating that the effect was dependent on the presence of relatively high S-(L + M) contrast. These results indicate that S-(L + M) signals impair spatial vision when combined with luminance. Since S-(L + M) signals are characterised by relatively large receptive fields, this is likely to be due to an increase in the size of the integration field over which contour-defining information is summed.

The visual system processes objects embedded in complex scenes that spatially vary in both luminance and colour. Luminance and colour signals thus constitute the building blocks from which perceived images are constructed. In this way, the processing of contrast sustains all subsequent, more complex visual processes. The human visual system has three channels that process initial inputs from our visual environment: a luminance channel which sums signals from Long- (L) and Middle-wavelength (M) cones, and two chromatic channels which oppose information from different cones. The S-(L + M) channel opposes the signals from the S-cones to a combination of L and M cone signals, while the L-M channel opposes the signals from the L and M cones themselves. These channels differ in their spatial and temporal resolutions-the S-(L + M) system in particular is characterised by larger receptive fields and slower response latencies^1^,^2^. Additionally there is evidence for sub-channels within these three opponent channels at the level of the lateral geniculate nucleus (LGN), as revealed via electrophysiological studies^3^ in the macaque and psychophysical masking in human observers^4^. Due to the speed with which luminance information is processed and the wide range of spatial frequencies that can be extracted from it, many theories of object recognition emphasise its contribution to the processing of object shape^5^,^6^. Indeed, the luminance channel has an abundance of oriented edge and line detectors suitable for shape processing^7^. Colour on the other hand is thought to contribute to scene analysis mostly through facilitating the segregation of surfaces that differ in chromaticity^8^.

But, although the luminance channel is more suitable for the extraction of object shape, it is not unlimited in its ability to resolve different shapes or features when they are presented within backgrounds. Extraction of shapes from backgrounds is commonly studied using contour integration paradigms. In these experiments, participants view a stimulus consisting of oriented Gabor patches and are asked to detect contours comprised of aligned patches embedded in approximately equally-spaced, randomly oriented patches^9^. Gaborised stimuli represent an ideal tool to study mid-level processes that underlie object recognition, such as figure-background segregation and perceptual grouping. Recently a stimulus set has been created that facilitates such study through providing Gaborised versions of line-drawings of objects from the Snodgrass and Vanderwart^10^ set, together with norms for different types of backgrounds and different tasks such as detection or identification^11^,^12^. Previously, studies that relied on Gabor fields with embedded contours demonstrated that colour and luminance rely on a common contour integration mechanism, performing roughly similarly across a wide range of contour curvatures^13^. Another phenomenon related to contour integration is crowding: a reduced ability to discriminate peripherally-presented objects or features (e.g., orientation) when they are situated near other objects or features^14^; this flanker-induced interference has been observed for other features as well, such as hue and saturation^15^. Crowding and contour integration are both thought to occur as a result of feature integration across space. The spatial extent of such integration is characterised in terms of “integration fields” (crowding) and “association fields” (contour integration). It has been suggested that the integration process and hence the spatial extents are the same in both phenomena^16^,^17^. In this way, crowding leads to ‘texturisation’ of features in the periphery^18^. Tripathy and Cavanagh^19^ investigated whether objects defined by chromatic signals are subject to the same crowding effect as those that are defined by luminance and found that that is indeed the case. Further, within channels, crowding seems to be specific to polarity; a green target is crowded more strongly by green flankers than by red flankers (colour pop-out is likely to contribute to this effect^20^). This was additionally demonstrated by Kennedy and Whitaker^21^, who found that performance for identifying the orientation of a single Gabor patch (the target) was degraded as the separation between it and a larger surrounding annulus (the flanker) was reduced, i.e., crowding occurred. This reduction in performance was similar when the contrast of the target and flanker were both modulated along the luminance, red-green or yellow-blue cardinal axes, however when the contrast of the target and flanker were modulated along different axes very little crowding occurred. However, there appears to be some individual variation, with some observers not exhibiting such selectivity^22^. Taken together, crowding among chromatic stimuli appears to be similar as that among non-chromatic stimuli.

Although both luminance and colour signals are subject to the rules that govern contour integration and crowding in a similar fashion, there are also some notable differences. In contour integration, luminance and colour differ in their sensitivities to various stimulus attributes, e.g. the spacing of contour elements-performance declines, for colour defined contours, more rapidly as element spacing increases^23^,^24^. Different orientation sensitivities of chromatic and luminance single edge detectors^25^,^26^ are also likely to provide a significant constraint for perceptual grouping into contours. So what would happen if colour and luminance are combined in a stimulus that requires contour integration? Previous work has examined integration of contours defined jointly by L-M and luminance contrasts where one of the contrasts was fixed and the other varied^25^. Contour integration was found to be sensitive to the sign of element contrast^27^, consistent with other processes such as crowding^19^ or global motion integration^28^. However, little is known about how chromatic and luminance channels interact when their signals are consistently combined and stimulus needs to be extracted from a background. In a contrast detection study, Wade^29^ found long-ranging surround suppression effects between S-cone and luminance contrasts. However, these were only present for temporally asynchronous stimuli with S-cone signals needing to precede luminance signals by about 40 ms for the effect to emerge. With simultaneous presentation, surround suppression mechanisms were largely independent. Thus it is not clear if interactions between chromatic and luminance signals should be expected in tasks that require discrimination of shapes that extend into the periphery and need to be extracted from a background. However, masking interactions between S-(L + M) and luminance signals reported by Wade^29^ could be expected to extend to such discrimination tasks, as masking has previously been reported to expand the range of crowding^30^.

Jennings and Martinovic^31^ demonstrated contrast-dependent interactions between luminance and chromatic signals, and additionally between colour pathways, i.e., when L-M and S-(L + M) signals were combined. These interactions occurred in a two-interval forced choice (2IFC) task which required participants to discriminate Gaborised images of familiar objects from unfamiliar shapes (‘non-objects’). But would the discrimination of shapes containing chromoluminant signals (i.e., containing both chromatic and luminance contrast) be affected by the presence of neighbouring background Gabor elements defined by the same type of chromoluminant contrast? In order to assess the effect of background on object classification based on different kinds of chromoluminant inputs, we first measured object discrimination thresholds with and without a sparse background for seven different combinations of chromatic and luminance signals, either isolating or combining the three visual channels (S-(L + M), L-M and L + M). We expected to replicate the findings of Jennings and Martinovic^31^ in the no-background condition. We also predicted a small increase in thresholds for conditions with backgrounds due to the relatively large spacing of Gabor elements, which should effectively minimise any crowding effects^32^. If the effect of background is uniform across different contrast-processing mechanisms, we will observe increases in threshold of similar size in each condition. However, if different types of contrast interact suppressively, due to the need to integrate information provided at different spatial scales and with different orientation sensitivities, then discrimination of objects embedded within backgrounds should lead to larger increases in threshold for conditions containing colour signals, in particular S-(L + M) signals, when compared to luminance alone.

## Results

### Experiment 1: The impact of background on the discrimination of objects defined by different colour/luminance content

**Figure 1:**
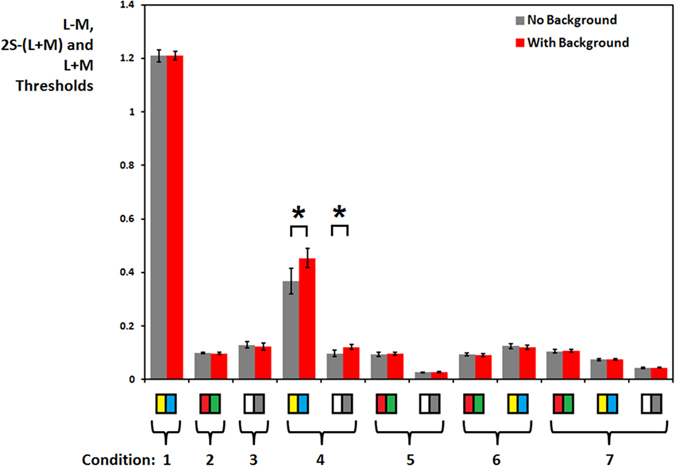
A comparison of object discrimination thresholds with (red bars) and without (grey bars) surrounding backgrounds. No significant differences exist, except when luminance is combined with a 2S-(L + M) signal (condition 4). Error bars represent ±2 standard errors.

Out of 15 participants, 8 were unable to achieve threshold in all of the conditions and were thus left out of the analysis. All of these participants failed to achieve threshold in one of the S-(L + M) conditions (with or without background, sometimes both), and often for several other conditions. Repeated measures ANOVAs were conducted on the remaining 7 participants in order to assess the effect of the presence of the background on the L-M, S-(L + M) and L + M contrast thresholds. The object discrimination thresholds are shown in Fig. 1. The abscissa is divided into subsections representing the seven luminance and chromatic conditions, as outlined in Table 1. Thresholds for each condition were subsequently decomposed into their individual chromatic and luminance components. For example, both the luminance and 2S-(L + M) components that compose condition 4, which combines luminance and S-(L + M) signals, have been plotted.

For the L-M signals, no effect of *background* was found (F(1, 6) = 0.41, p = 0.54), however an effect of *chromatic/luminance contrast combinations* was significant (F(3, 18) = 11.01, p < 0.001, ηp^2^ = 0.65). No interaction between *background* and *chromatic/luminance contrast combination* existed (F(3, 18) = 0.71, p = 0.56). The main effect of colour/luminance contrast combination was tested using Bonferroni-corrected paired t-tests, with the significance level dropped to 0.008 as 6 comparisons were conducted. There were no differences between isolated thresholds and thresholds combined with luminance (t(6) = 0.17, p = 0.87) and S-(L + M) signals (t(6) = 0.63, p = 0.56). L-M contrast at threshold was however significantly lowered for the full combination (t(6) = –11.99, p < 0.001). Contrasts did not differ between the combination with luminance and with S-(L + M) (t(6) = 0.46, p = 0.66) or combination with luminance and the full combination (t(6) = –3.42, p = 0.014; this is equivalent to a trend of p = 0.084), but were significantly different between the combination with S-(L + M) and the full combination (t(6) = –4.54, p = 0.004).

For the S-(L + M) signals a significant effect of adding the *background* (F(1, 6) = 10.49, p = 0.018, ηp^2^ = 0.64) and also a significant effect of *chromatic/luminance contrast combinations* (F(3, 18) = 6773.19, p < 0.001, ηp^2^ = 1.00) was found; additionally a significant interaction existed (F(1.20, 7.22) = 6.13, p = 0.038, ηp^2^ = 0.51). The main effect of colour/luminance contrast combination was tested using Bonferroni-corrected paired t-tests, with the significance level dropped to 0.008 as 6 comparisons were conducted. The effect was such that contrast present at threshold was lower for combined conditions than for the isolating conditions (isolating vs. luminance combination: t(6) = 75.91, p < 0.001; isolating vs. L-M combination: t(6) = 118.50, p < 0.001; isolating vs. full combination: t(6) = 111.35, p < 0.001). There was also significantly more contrast present at threshold for combination with luminance than for the combination with L-M (t(6) = 27.06, p < 0.001) or for the full combination of contrasts (t(6) = 36.21, p < 0.001). These last two also differed (t(6) = 13.12, p < 0.001). The main effect of the background was such that thresholds were elevated in its presence, in line with the hypotheses. Post-hoc tests for the interaction were targeted at the effect of the background, assessing if it was present in all colour/luminance conditions. The level of significance was dropped to 0.0125 as 4 comparisons were made. There was a trend for an increase in contrast at threshold for the combination with luminance (t(6) = −2.85, p = 0.029), all other comparisons were not significant (S-(L + M) isolating: t(6) = −0.86, p = 0.42; combination with L-M: t(6) = 1.10, p = 0.32; all signals combined: t(6) = −0.57, p = 0.59). To increase the power of these tests, we conducted them again, but including all the participants with sufficient data. Whilst there was now a significant increase in threshold when S-(L + M) signals were combined with luminance (t(12) = −3.13, p = 0.009), this was not the case in other combinations (with L-M: t(11) = 1.32, p = 0.21; full: t(11) = −0.36, p = 0.73).

For the L + M signals there was no effect of adding the *background* (F(1, 6) = 1.83, p = 0.23). There was a significant effect of *chromatic/luminance contrast combinations* (F(1.44, 8.62) = 247.33, p < 0.001, ηp^2^ = 0.98); additionally a significant interaction with the presence of the background existed (F(3, 18) = 4.79, p = 0.013, ηp^2^ = 0.44). The main effect of colour/luminance contrast combination was tested using Bonferroni-corrected paired t-tests, with the significance level dropped to 0.008 as 6 comparisons were conducted. There was significantly less luminance contrast at threshold for the combinations with L-M (t(6) = 20.02, p < 0.001) and the full combination (t(6) = 13.88, p < 0.001), but the difference was not significant for the combination with S-(L + M) (t(6) = 2.81, p = 0.03). Likewise, there was also less contrast for the combination with L-M than with S-(L + M) (t(6) = 28.79, p < 0.001) and for the full combination than with S-(L + M) (t(6) = 23.31, p < 0.001). Finally, there was more contrast for the full combination than for the combination with L-M (t(6) = −15.09, p < 0.001). Post-hoc tests of the interaction were again targeted at whether the effect of the background was specific to any colour/luminance combinations, with the criterion p-value dropped to 0.0125. The tests were inconclusive (isolating: t(6) = 0.75, p = 0.49; with S-(L + M): t(6) = −2.82, p = 0.03; with L-M: t(6) = −1.37, p = 0.22; full: t(6) = −0.54, p = 0.61). In order to increase the power of these tests, we conducted them again, but including all the participants with sufficient data in those conditions, not only those included in the overall ANOVAs. Whilst there was a significant increase in threshold when S-(L + M) signals were combined with luminance (t(12) = −3.11, p = 0.009), this was not the case in other combinations (isolated: t(11) = −0.04, p = 0.97; with L-M: t(11) = −0.98, p = 0.35; full: t(11) = −0.33, p = 0.75).

The observed effects of the *colour/luminance contrast combination* reproduce previous findings^31^. For example, comparing the luminance and L-M signals in isolation to the same signals when contrasts were presented in combination, there is less luminance contrast and the same amount of L-M contrast, reproducing the result that L-M is driving the discrimination in the presence of a relatively low luminance signal. Comparing luminance in isolation to luminance combined with an S-(L + M) signal, no significant difference is found between luminance levels but significantly less S-(L + M) signal is present, reproducing the result that luminance drives the discrimination when combined with S-(L + M). S-(L + M) was confirmed to be the least effective in sustaining performance in the object discrimination task, as it only ever drove performance when isolated; it also proved to be the most difficult condition for our generally inexperienced participants, leading to a reduced final sample size. Finally, a facilitative interaction between signals was observed when all the signals were combined, as contrast at threshold in each of them was lower than when isolated.

Adding the background did cause an increase in thresholds, but they were largely restricted to stimuli that contained S-(L + M) signals, in particular in combination with luminance. This was followed up in the second experiment, which aimed to identify the dependence of the background effect on (1) the amount of S-(L + M) signal in the stimulus, and (2) the density of the Gabor elements.

### Experiment 2: Impact of background density on the discrimination of objects defined by different S-cone/luminance content

The S-(L + M) isolating condition again proved challenging for our participants, especially when it was joined with a background, so that only 7 and 3 observers achieved 75% correct with the sparse and dense backgrounds, respectively. For the luminance-isolating and S-(L + M) combined with luminance at both 30° and 60° elevations, 18 out of the 21 participants produced usable data in all of the conditions. Therefore, we focused our analysis on these three conditions, as they are sufficient to answer outstanding questions on the dependence of the background effect on the amount of S-(L + M) signal in the stimulus and the density of the Gabor elements.

**Figure 2:**
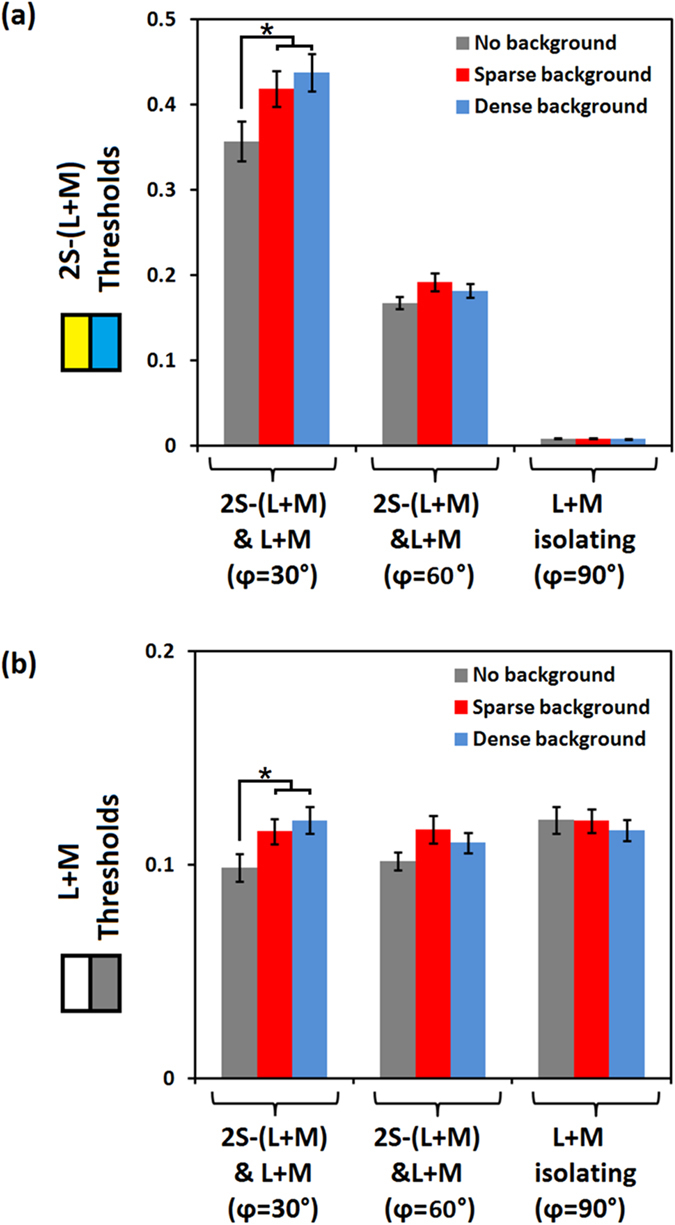
Contrasts required for threshold. Thresholds are shown in (**a**) for 2S-(L + M) and (**b**) for L + M for the conditions that combine 2S-(L + M) and luminance at 30° and 60° DKL luminance elevations, as well as the luminance isolating condition. Error bars represent (±)2 standard errors.

A repeated measures ANOVA was conducted to assess the effect different *backgrounds* (none, sparse and dense) had on the L + M signals at threshold when presented with different *levels of S-(L + M) signal* (none, low, high). The amount of S-(L + M) signal was manipulated by collecting data when luminance was isolated, and when it was defined with a luminance elevation of either 30° or 60° which additionally combined it with an S-(L + M) signal. The S-(L + M) threshold data is presented in Fig. 2(a), the bars are grouped by luminance elevation, and within each triplet of bars the background condition is illustrated, Fig. 2(b) is of the same format but plots the L + M contrasts. The ANOVA showed an effect of *background* and of *chromatic/luminance co*m*bination* (F(2, 34) = 3.78, p = 0.033, ηp^2^ = 0.18 and F(2, 34) = 3.83, p = 0.032, ηp^2^ = 0.18 respectively), while the interaction was verging on significance (F(4, 68) = 2.12, p = 0.057, ηp^2^ = 0.13). Post-hoc tests for the main effect of background (criterion p-value of 0.017) showed that whilst thresholds were increased for the sparse background (t(17) = −2.18, p = 0.011), this was not the case for the dense background (t(17) = −2.18, p = 0.043). The two background levels did not differ (t(17) = 0.41, p = 0.69). Post-hoc tests for the main effect of colour/luminance combination (criterion p-value of 0.017) showed that the amount of luminance needed to achieve threshold was equal in all three conditions (30° vs. 60° elevation: t(17) = 0.59, p = 0.57; 30° vs. isolated: t(17) = −2.18, p = 0.044, 60° vs. isolated: t(17) = −2.58, p = 0.02); the last effect between 60° elevation and isolated luminance would qualify as a trend equivalent to p = 0.06, and this is probably what drove the main effect that we observed. Post-hoc tests were also conducted to assess the potential source of the trend for an interaction between background and S-(L + M) contrast level, by comparing all conditions without the background with conditions with background (criterion p-value of p = 0.008). The tests indicated a significant difference between the no background with a sparse background when S-(L + M) signals were combined with luminance at an elevation of 30° (t(17) = −3.42, p = 0.003), and a trend for the dense background at the same elevation (t(17) = −2.79, p = 0.013, equivalent to p = 0.078). Other comparisons were not significant (none vs. sparse at 60°: t(17) = −2.06, p = 0.056; none vs. dense at 60°: t(17) = −1.50, p = 0.15; none vs. sparse at 90°: t(17) = 0.04, p = 0.97; none vs. dense at 90°: t(17) = 0.77, p = 0.45). In order to increase the power of these tests, we conducted them again, but including all the participants with sufficient data in those conditions. There was a significant increase in threshold for both sparse (t(19) = −3.50, p = 0.002) and dense backgrounds (t(19) = −3.23, p = 0.004) at 30° elevation, but this was not the case in other combinations (none vs. sparse at 60°: t(20) = −1.46, p = 0.16; none vs. dense at 60°: t(17) = −1.66, p = 0.11; none vs. sparse at 90°: t(19) = −0.26, p = 0.80; none vs. dense at 90°: t(2) = 0.94, p = 0.36).

## Discussion

We assessed if the presence of a surrounding background has an effect on discriminating objects defined by different colour/luminance content. Unexpectedly, thresholds were elevated in the presence of a background only when objects were defined with a luminance signal combined with an S-(L + M) signal. This effect was found to depend on the relatively large amount of S-(L + M) signal being present, but did not increase when the background was made denser. Our results are consistent with reports of interactions between S-(L + M) and luminance signals that led to cross-channel masking over a wider spatial range than for luminance signals alone^27^. Such increased masking is likely to have affected other processes that require integration of information in extra-foveal spatial fields, in line with the observation that an increased extent of crowding is observed in the presence of weak masking. In conclusion, the object discrimination costs we observed are likely to occur due to compromised contour integration and increased crowding, with the possible mechanism being S-cones’ larger receptive fields offering a larger integration window for co-localised luminance information.

The effects we observe are consistent with Wade’s^29^ observation of relatively long-range suppression between luminance and S-cone signals, but here we demonstrate that such effects can also be observed with long-duration stimuli in an object discrimination task. Performance in the object discrimination task for combined L + M and S-(L + M) signals is driven by the luminance content of the stimulus, as there are no significant differences between the luminance content from when luminance alone is present. Thus, it is possible that the long duration of the stimulus gave the opportunity for temporally lagged suppressive effects of S-cone signals to emerge. There is considerable evidence that the spatial interaction between objects in crowding extends to about half the eccentricity of the target, irrespective of the type of object. This has been called Bouma’s law^14^,^32^. However, there are a few exceptions to this law, where mild masking of the target^30^ or inducing grouping^33^ can dramatically change the spatial extent of crowding. It is possible that due to the effects of good continuation along the contour in the object discrimination task, the suppressive effect of S-(L + M) inputs requires a relatively large amount of contrast in order to be seen, as grouping has the capability of ‘uncrowding’^33^. This grouping effect of good continuation could also explain why we did not observe the adverse effect of background in any other conditions, with the only significant differences being brought about by combining S-(L + M) contrast with luminance. Last but not least, the unexpected lack of increase in threshold for higher Gabor density could also be associated to grouping, as the denser background may have been subject to stronger grouping into a separate, texture-like background. As we did not predict such an effect, this would need to be examined in future experiments. However, the S-(L + M) isolating condition did prove challenging to a large number of participants, especially with the increase from sparse to dense background in the second experiment. Therefore, the adverse effects of the background could also be present in the S-(L + M) isolating condition, but fail to emerge due to a rapid loss of ability to discriminate S-(L + M) defined large-scale, complex stimuli that extend into the periphery and are further surrounded with backgrounds whilst maintaining central fixation.

Considered jointly, our results indicate that S-(L + M) signals interact suppressively with luminance signals in spatial vision tasks and that this most likely induces a failure to correctly perceive the outer contour of the stimulus. This could be due to compromised contour integration and/or crowding. In fact, crowding is anisotropic, so that the outer flanker enacts a stronger influence than the inner flanker^34^, which is consistent with the appearance of our stimuli, with backgrounds added to the outside.

Previously, S-(L + M) mechanisms were considered to be mainly relevant for colour appearance, as their low spatial and temporal resolution make them ill-suited for spatial vision tasks when presented in isolation (for a review, see Shevell & Kingdom^35^). However, even in colour appearance, S-cone signals have been known to interact with L and M cone signals^36^ and their processing is now known to involve multiple, complex mechanisms already at the early levels of processing^3^. We suggest that the potential effect of S-cone signals on perceptual organisation processes and subsequently object discrimination could occur due to their involvement in texturisation-as S-cone receptive fields are larger they could provide a low-level mechanism that pools information over long distances. Thus, S-cone signals could play a role in creating a framework in which shape and object perception driven by mechanisms driven by L and M cone signals can operate. We conclude that S-cones are likely to play a role in spatial vision after all, albeit when co-stimulated with the luminance channel to a sufficient level, and may contribute to a range of mid-level vision, figure/background processes by providing a larger integration window for higher-acuity co-localised luminance signals.

## General Methods

### Participants

15 naive, inexperienced observers took part in experiment 1 (2 male, 18–40 years, mean age 23.5 years), and 21 naive, inexperienced observers took part in experiment 2 (17 male, 18–27 years, mean age 21.6). No observers from experiment 1 participated in experiment 2. All observers reported normal or corrected-to-normal visual acuity and had adequate colour vision as assessed with the Cambridge Colour Test^37^ (CCT). The study was approved by the institutional *Psychology Ethics Committee* of the School of Psychology, University of Aberdeen. Psychophysical testing was carried out in accordance with the guidelines given in the Declaration of Helsinki, which the committee subscribes to; prior to the start of each experiment each observer gave written (signed) informed consent.

### Stimuli

**Figure 3:**
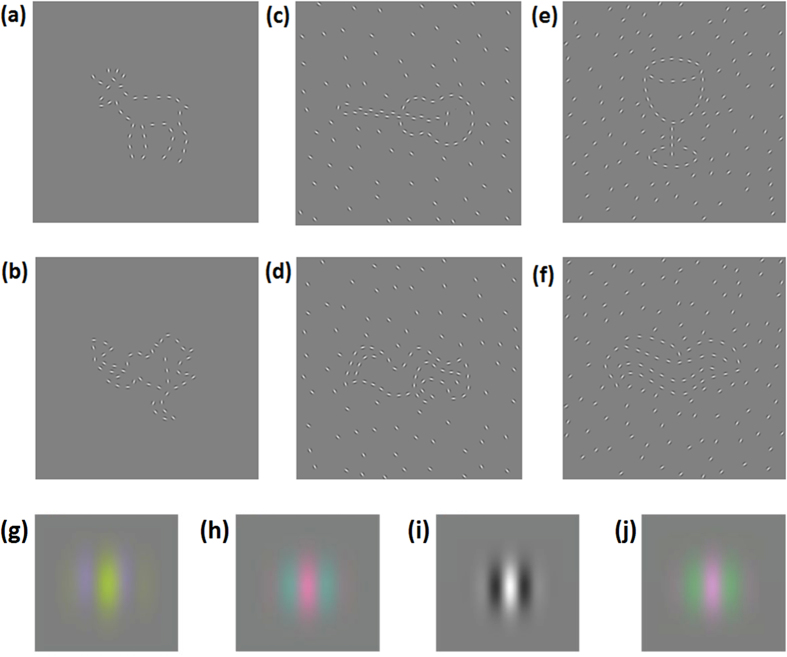
Examples of stimuli and individual colour and luminance defined Gabor patches. Nameable objects (top row) and unnameable non-objects (bottom row) are embedded within different background types; no background (**a**) and (**b**), a sparse background (**c**) and (**d**), and a dense background (**e**) and (**f**). Example Gabor patches are illustrated in (**g–j**), these correspond respectively to conditions 1, 2, 3 and 6 shown in Fig. 1, i.e., S-(L + M) isolating, L-M isolating, luminance isolating and L-M combined with S-(L + M), respectely.

The stimulus set from Jennings and Martinovic^31^ was used for experiments 1 and 2. This set consists of 377 nameable, familiar objects and 377 matching unnameable, unfamiliar objects (also known as “non-objects”) and is similar to the image library provided by Sassi *et al*.^12^. The stimuli were composed of a series of centre-symmetric 3 cpd Gabor patches. This spatial frequency was chosen so that roughly equal contrast dependence of orientation sensitivity across channels would be maintained^28^. The object/non-object stimuli were created by selecting suitable line images of familiar, nameable objects from various stimulus sets^38^,^39^,^40^ and also by the manual digital drawing of additional line images. The corresponding non-object images were created by distorting the line images of the objects until they became unrecognisable; this was achieved using image processing software (GNU Image Manipulation Software). The lines of these images were replaced with a series of Gabor patches with the position and orientation of each Gabor patch predefined by hand in order to ensure that shape-defining lines were distributed along the relevant parts of the images, such as corners and T-junctions. Figure 3 shows an example of (a) an object and (b) a non-object, respectively. The process of scrambling the object images into non-object images was conducted in a way that preserved some important attributes of the initial object images, including the visual complexity of the images as reflected in jpeg file size (for a similar approach to complexity, see Szekely & Bates^41^) and their aspect ratio. Non-objects were additionally constrained to have a closed outer contour in order be consistent with that property of objecthood and preventing them from appearing as random clusters of Gabor patches. The objects and non-objects were piloted in a naming task and only stimuli that were reliably identified, i.e., either named correctly by a majority of the participants or declared to be non-objects, were included in the stimulus set. On average, the final set of object stimuli subtended a width and height of 6.7 ± 1.1 and 2.9 ± 1.0 degrees (mean ± SD), respectively, whilst the non-object stimuli subtended a width and height of 7.6 ± 0.9 and 2.8 ± 0.8 degrees (mean ± SD), respectively. Gabor patches for 4 colour and luminance conditions are illustrated in Fig. 3g–j, for more details on the Gabor properties and the attributes of the stimulus set, see Jennings and Martinovic^31^.

Experiments 1 and 2 employed this object/non-object stimulus set, with the addition of semi-random backgrounds. These backgrounds were composed of Gabor patches that were randomly positioned except for the following constraint: the patches could only be located to the exterior of the objects, with a minimum distance of 0.15° to the outer contour of the object/non-object. This constraint was set as our object and non-object images could also have inner features. The addition of random Gabors within the image would thus compromise discrimination of these inner features. For each stimulus the background Gabor patches were aligned at either ±45° orientation, with ±20° of random orientation jitter. These relatively uniform backgrounds were employed as typically in natural scenes, especially indoor and urban environments, the scenes background has a non-random structure. Experiment 1 employed a sparse background Gabor patch density (0.44 Gabor patches per square degree of visual angle); experiment 2 also employed this background density and an additional, 30% denser background. Figure 3c–f shows examples of objects and non-objects embedded within the sparse and dense backgrounds. All stimuli were displayed on a mid-grey background which was located at CIE xyY = (0.29, 0.32, 51.6 cd m^−2^).

### Stimulus generation

**Figure 4:**
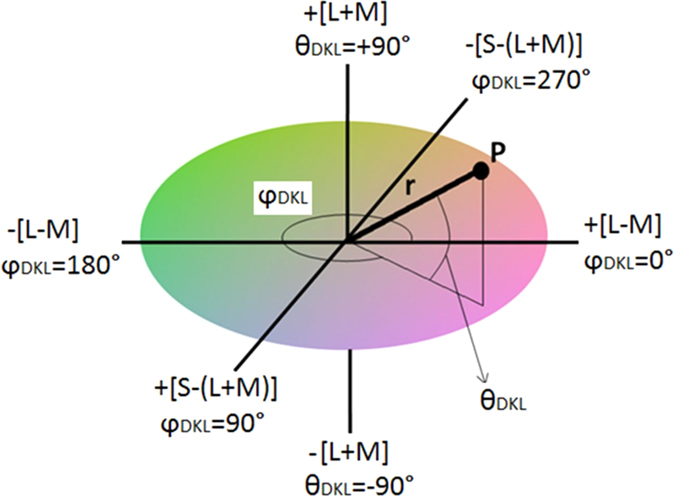
The DKL colour space. The horizontal surface represents the isoluminant plane containing only chromatic variations along the L-M and S-(L + M) axes. Luminance (L + M) increases and decreases along the positive and negative parts of the vertical axis, respectively. Point P represents a chromaticity with both L-M and S-(L + M) components and luminance (L + M), these are defined by: 1) radius (r) which defines its distance from the centre of the space (whitepoint), 2 hue angle or angle of rotation (φ_DKL_), which defines the chromaticity, and 3) luminance angle or angle of elevation (θ_DKL_), which defines the amount of luminance present in the stimulus, with +90° elevation being an achromatic stimulus lighter than the background and a –90° elevation being an achromatic stimulus darker than the background.

The Derrington Krauskopf and Lennie (DKL) colour space^42^ was used to describe the chromatic and luminance properties of the stimuli. Figure 4 shows a representation of the DKL colour space indicating the two chromatic (L-M and S-(L + M)) axes and the luminance axis (L + M), along with a vector (P) defining a particular chromaticity and luminance with a given DKL radius (r), chromatic angle ϕ_DKL_, and luminance elevation θ_DKL_. The stimuli chromatic and luminance components were defined so as to excite the L-M (reddish-greenish), S-(L + M) (yellowish-bluish) or L + M (luminance) channels either in isolation or in various combinations; the two chromatic pathways combined and L-M and S-(L + M) combined, separately, with an L + M luminance signal, and finally all three types of signals combined, examples of these are depicted in Fig. 3g–j. In DKL space, when chromatic signals are combined with luminance signals this can be done at different degrees of elevation (θ_DKL_). Higher DKL elevation leads to more luminance being present in a stimulus of the same radius (see Jennings and Martinovic^31^ for more detail).

Stimuli were produced using a dedicated visual stimulus generator (ViSaGe; CRS, UK). The stimuli were displayed on a calibrated 21'' Viewsonic P227f CRT Monitor controlled by the ViSaGe system. The display was gamma corrected based on measurements acquired with a ColorCAL MKII colorimeter (CRS, UK) and look-up tables were generated based on measurements of the spectral power distributions of the CRT’s red, green and blue phosphors, acquired using a Spectroradiometer (SpectroCAL, CRS, UK) and the cone fundamentals^43^,^44^. Colour Toolbox (CRS, UK) was used to implement the DKL space^45^ and the CRS Toolbox (CRS, UK) for MatLab (Mathworks, USA) was used to run the experiment.

### Heterochromatic flicker photometry (HCFP)

Individual differences in the luminous efficiency function^46^ can result in a small, but significant, luminance signal being present within isoluminant chromatic signals. Heterochromatic flicker photometry^47^ (HCFP) was used to measure luminance correction factors for individual observers, following a previously outlined procedure^28^,^48^. HCFP utilizes the difference in the temporal resolution of the luminance pathway compared to the chromatic pathway, the luminance pathway being capable of higher temporal resolution and hence able to process higher frequency flicker. Therefore, if a subject can adjust the relative amounts of luminance within a 20 Hz chromatically flickering display so that the perception of flicker is eliminated or at least minimised the luminance difference will also be minimized providing an isoluminant correction for individuals. In the DKL space, this correction can be realised for individual observers by tilting the DKL isoluminant plane. The three isoluminant conditions used in this study were L-M in isolation which corresponds to θ_DKL_ = 0 and 180° (reddish and greenish axis), S-(L + M) in isolation which corresponds to θ_DKL_ = 90 and 270° (yellowish and bluish axis), and both L-M and S-(L + M) combined which corresponds to θ_DKL_ = 135 and 315° (magenta and a yellowish-green direction). The spatial attributes of the flickering stimuli were chosen from experimental stimuli randomly, and comprised of objects or non-objects with or without backgrounds. All observers reported that by adjusting the luminance levels they could find a setting where the flickering stimulus became minimal or completely nulled for each of the conditions. Each observer repeated this luminance adjustment task eight times for each condition, the highest and the lowest values were removed as outliers and a mean correction was determined from the remaining six observer adjustments.

### Procedure

Threshold measurements using a 2IFC procedure were performed. The observers’ task was to identify which interval contained the object. Two staircases for each of the colour/luminance combinations were run together in one testing block for a specific type of background, each staircase modulating the individual Gabor patches’ contrast. In experiment 1, each staircase consisted of 25 trials and the seven colour/luminance conditions were tested with a sparse background and no background present. In experiment 2, each staircase consisted of 29 trials and 4 combinations of colour/luminance were tested without a background, with a sparse background or with a dense background. The chromatic and luminance conditions for each experiment are outlined in Table 1. The number of trials per condition was limited by the overall number of stimulus items in the set, as we did not want stimulus repetitions (see Jennings and Martinovic^31^ for a similar approach). Within each trial the first stimulus was presented for 1200 ms, followed by 1000 ms of fixation, followed by the 1200 ms of second stimulus. After the second stimulus was displayed the fixation cross remained on screen and the system waited until a response was given by the observer before starting the next trial. The observers were instructed to guess if they were unable to determine which interval contained the object. No feedback on accuracy was given during the experiment in order to minimise explicit learning of stimulus category characteristics. Observers’ responses controlled the contrast of the stimuli in an adaptive fashion. Logistic functions were fitted to the data to obtain the DKL radius (r) that led to 75% accuracy of discrimination for each condition. The Palamedes Toolbox^49^ was used for running the staircases and for the fits. Thresholds in terms of *r* were then transformed into a triplet of contrast values representing the contrast in the two chromatic and luminance pathways (see the data processing section for details).

Experiments were conducted over several sessions per subject. The first session always contained the Cambridge Colour Test and the heterochromatic flicker photometry. Subsequent sessions lasted 1 hour at most. The order of the blocks with different background types was approximately balanced across the sample in Experiments 1 and 2. As the data was to be analysed using repeated measures ANOVAs only subjects that obtained psychometric functions that straddled the 75% accuracy point for every condition were included in the analysis. Observers that did not reach a threshold of 75% accuracy in were re-tested for that particular condition. For experiment 1, there was one re-test on average per observer that did not provide a reliable threshold measurement. For experiment 2, multiple retests were required for the S-(L + M) isolating conditions and these were later excluded from analysis due to lack of data, as thresholds could not be reached for a relatively large number of participants. For the combinations of colour and luminance at elevations of 30°, 60° and for the luminance isolating conditions (elevation of 90°) an average 0.59, 0.06 and 0.35 retests per observer were required over the no background, sparse and dense background conditions, respectively.

### Data processing

Not all observers could achieve a 75% correct performance in every condition even after re-tests. Their data were excluded from the analysis. The S-(L + M) isolating condition proved the most challenging, similarly to Jennings and Martinovic^31^. In experiment 1, this resulted in only 7 observers’ data being included in the repeated measures ANOVA, as we needed data from all conditions in order to test our hypotheses. In Experiment 2, once again, the S-(L + M) isolating condition proved difficult, which was especially the case in the presence of the background. Therefore, we excluded the S-(L + M) isolating condition from the ANOVA, and compared performance in the luminance isolating condition and the two different combinations of S-(L + M) signals with luminance instead.

The measured thresholds expressed in terms of DKL radius (r), chromatic angle (ϕ_DKL_) and luminance elevation (θ_DKL_) were converted into L-M, S-(L + M) and L + M mechanism contrasts. This was achieved by converting the CIE 1931 xyY coordinates of each condition’s threshold into L-, M- and S-cone excitations. The conversion of CIE XYZ values into L-, M- and S-cone excitations was achieved using a 3 × 3 transformation matrix which was derived according to the method outlined in Macleod and Golz^50^. The Stockman-Sharpe cone fundamentals^43^,^44^ along with the measured red, green and blue spectral power distributions from the Viewsonic P227f CRT Monitor were used as inputs.

The Michelson cone contrasts (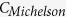) required for threshold were calculated according to Equation. 1, where 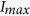 and 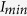 represent the maximal and minimal L, M and S-cone contrasts of the Gabor patches at threshold. L-M, S-(L + M) and L + M contrasts were calculated from such Michelson cone contrasts.


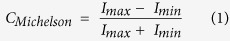


Repeated measures ANOVAs were used to analyse the data. Experiment 1 had the factor of *background* (none or sparse) and the factor of *chromatic/luminance contrast combination*, with 4 levels for chromatic contrasts (isolating contrast, combination with luminance, combination with the other chromatic contrast, combination of all three contrast types) and 4 levels for luminance contrast (isolating contrast, combination with S-(L + M), combination with L-M, combination of all three contrast types). The Greenhouse-Geisser correction of the degrees of freedom was used when the assumption of sphericity was violated. Experiment 2 had the factor of *background* (none, sparse and dense) and the factor of *S-(L + M)* or *luminance contrast combination*, each with 3 levels (isolating, combination at DKL elevation angle of 30° and combination at DKL elevation angle of 60°). Bonferroni corrected post-hoc t-tests were performed when necessary.

## Additional Information

**How to cite this article**: Jennings, B. J. *et al*. Combining S-cone and luminance signals adversely affects discrimination of objects within backgrounds. *Sci. Rep.* **6**, 20504; doi: 10.1038/srep20504 (2016).

## Figures and Tables

**Table 1:** Summary of the chromatic and luminance conditions employed in each experiment, a tick denotes the condition was tested.

Condition number	Mechanism(s) stimulated	Luminance Elevation (φ; in degrees)	Experiment	
1	2			
1	S-(L + M)	0	X	
2	L-M	0	X	X
3	L + M	90	X	X
4	S-(L + M) & L + M	30	X	X
5	L-M & L + M	30	X	
6	L-M & S-(L + M)	0	X	
7	L-M & S-(L + M) & L + M	30	X	
8	S-(L + M) & L + M	60		X

Condition numbers correspond to those given in Figure 1.

## References

[b1] Stockman A. & Brainard D. H. Color vision mechanisms. In Bass M. (Ed.), OSA Handbook of Optics (3rd edition) (pp. 11.11-11.104). New York: McGraw-Hill (2010).

[b2] Smithson H. E. S-cone psychophysics. Visual Neurosci 31, 211–225 (2014). 10.1017/S0952523814000030 24759446

[b3] Tailby C., Solomon S. G. & Lennie P. Functional asymmetries in visual pathways carrying S-cone signals in macaque. J Neurosci 9, 4078–4087 (2008). 18400907 10.1523/JNEUROSCI.5338-07.2008 PMC2602833

[b4] Sankeralli M. J. & Mullen K. T. Bipolar or rectified chromatic detection mechanisms? Visual Neurosci 18, 127–135 (2001). 10.1017/s0952523801181125 11347810

[b5] Bar M. A cortical mechanism for triggering top-down facilitation in visual object recognition. J Cogn Neurosci 15, 600–609 (2003). 12803970 10.1162/089892903321662976

[b6] Sowden P. T. & Schyns P. G. Channel surfing in the visual brain. Trends Cogn Sci 10, 538–545 (2006). 17071128 10.1016/j.tics.2006.10.007

[b7] Hesse G. S. & Georgeson M. A. Edges and bars: where do people see features in 1-D images? Vis Res 45, 507–525 (2005). 15610754 10.1016/j.visres.2004.09.013

[b8] Gegenfurtner K. R. & Rieger J. Sensory and cognitive contributions of color to the recognition of natural scenes. Curr Biol 10, 805–808 (2000). 10898985 10.1016/s0960-9822(00)00563-7

[b9] Field D. J., Hayes A. & Hess R. F. Contour integration by the human visual-system-evidence for a local association field. Vis Res 33, 173–193 (1993). 8447091 10.1016/0042-6989(93)90156-q

[b10] Snodgrass J. G. & Vanderwart M. A standardized set of 260 pictures: Norms for name agreement, image agreement, familiarity, and visual complexity. J Exp Psychol-Learn Mem Cogn 6, 174–215 (1980). 10.1037//0278-7393.6.2.174 7373248

[b11] Sassi M., Machilsen B. & Wagemans J. Shape detection of Gaborized outline versions of everyday objects. Iperception, 3(10), 745–764 (2012). 23483752 10.1068/i0499 PMC3589903

[b12] Sassi M., Vancleef K., Machilsen B., Panis S. & Wagemans J. Identification of everyday objects on the basis of Gaborized outline versions. Iperception 1, 121–142 (2010). 23145218 10.1068/i0384 PMC3485765

[b13] Mullen K. T., Beaudot W. H. A. & McIlhagga W. H. Contour integration in color vision: a common process for the blue-yellow, red-green and luminance mechanisms? Vis Res 40, 639–655 (2000). 10824267 10.1016/s0042-6989(99)00204-7

[b14] Bouma H. Interaction effects in parafoveal letter recognition. Nature 226, 177–8 (1970). 5437004 10.1038/226177a0

[b15] van den Berg R., Roerdink J. B. T. M. & Cornelissen F. W. On the generality of crowding: Visual crowding in size, saturation, and hue compared to orientation. J Vis 7(14) (2007). 10.1167/7.2.14 18217829

[b16] May K. A. & Hess R. F. Ladder contours are undetectable in the periphery: A crowding effect? J Vis 7 (2007). 10.1167/7.13.9 17997637

[b17] Chakravarthi R. & Pelli D. G. The same binding in contour integration and crowding. J Vis 11, 1–12 (2011). 10.1167/11.8.10 PMC3624759 21757504

[b18] Parkes L., Lund J., Angelucci A., Solomon J. A. & Morgan M. Compulsory averaging of crowded orientation signals in human vision. Nat Neurosci 4, 739–44 (2001). 11426231 10.1038/89532

[b19] Tripathy S. P. & Cavanagh P. The extent of crowding in peripheral vision does not scale with target size. Vis Res 42, 2357–2369 (2002). 12350424 10.1016/s0042-6989(02)00197-9

[b20] Gheorghiu E. & Kingdom F. A. A. Chromatic properties of texture-shape and of texture-surround suppression of contour-shape mechanisms. J Vis 12 (2012). doi: 10.1167/12.6.16. 22693334

[b21] Kennedy G. J. & Whitaker D. The chromatic selectivity of visual crowding. J Vis 10 (2010). doi: 10.1167/10.6.15. 20884564

[b22] Kooi F. L., Toet A., Tripathy S. P. & Levi D. M. The effect of similarity and duration on spatial interaction in peripheral-vision. Spatial Vision 8, 255–279 (1994). 7993878 10.1163/156856894x00350

[b23] Beaudot W. H. A. & Mullen K. T. Processing time of contour integration: the role of colour, contrast, and curvature. Perception 30, 833–853 (2001). 11515956 10.1068/p3164

[b24] Beaudot W. H. A. & Mullen K. T. How long range is contour integration in human color vision? Visual Neurosci 20, 51–64 (2003). 10.1017/s0952523803201061 12699083

[b25] McIlhagga W. H. & Mullen K. T. Contour integration with colour and luminance contrast. Vis Res 36, 1265–1279 (1996). 8711906 10.1016/0042-6989(95)00196-4

[b26] Wuerger S. M. & Morgan M. J. The input of the long- and medium wavelength sensitive cones to orientation discrimination. JOSA A 16, 436–442 (1999).

[b27] Yeshurun E. & Rashal Y. Contrast dissimilarity effects on crowding are not simply another case of target saliency. J Vis 14 (2014). doi: 10.1167/14.6.9. 25476716

[b28] Wuerger S. M., Ruppertsberg A., Malek S., Bertamini M. & Martinovic J. The integration of local chromatic motion signals is sensitive to contrast polarity. Visual Neurosci 28, 239–246 (2011). 10.1017/S0952523811000058 21426617

[b29] Wade A. R. Long-range suppressive interactions between S-cone and luminance channels. Vis Res 49, 1554–1562 (2009). 19344735 10.1016/j.visres.2009.03.023 PMC2703610

[b30] Vickery T. J., Shim W. M., Chakravarthi R., Jiang Y. H. V. & Luedeman R. Supercrowding: Weakly masking a target expands the range of crowding. J Vis 9 (2009). doi: 10.1167/9.2.12. 19271922

[b31] Jennings B. J. & Martinovic J. Luminance and color inputs to mid-level and high-level vision. J Vis 14 (2014). doi: 10.1167/14.2.9. 24520151

[b32] Pelli D. G. & Tillman K. A. The uncrowded window of object recognition. Nat Neurosci 11, 1129–1135 (2008). 18828191 10.1038/nn.2187 PMC2772078

[b33] Manassi M., Sayim B. & Herzog M. H. When crowding of crowding leads to uncrowding. J Vis 13 (2013). doi: 10.1167/13.13.10. 24213598

[b34] Petrov Y., Popple A. V. & McKee S. P. Crowding and surround suppression: Not to be confused. J Vis 7 (2007). doi: 10.1167/7.2.12. PMC2361428 18217827

[b35] Shevell S. K. & Kingdom F. A. A. Color in complex scenes. Annu Rev Psychol 59, 143–166 (2008). 18154500 10.1146/annurev.psych.59.103006.093619

[b36] Knoblauch K. & Shevell S. K. Relating cone signals to color appearance: Failure of monotonicity in yellow/blue. Visual Neurosci 18, 901–906 (2001). 10.1017/s0952523801186062 12020080

[b37] Regan B. C., Reffin J. P. & Mollon J. D. Luminance noise and the rapid determination of discrimination ellipses in color deficiency Vis Res 34, 1279–1299 (1994). 8023437 10.1016/0042-6989(94)90203-8

[b38] Alario F. X. & Ferrand L. A set of 400 pictures standardized for French: Norms for name agreement, image agreement, familiarity, visual complexity, image variability, and age of acquisition. Behav Res Meth Ins C 31, 531–552 (1999). 10.3758/bf03200732 10502875

[b39] Bates E., D’Amico S., Jacobsen T., Szekely A., Andonova E., Devescovi A. . Timed picture naming in seven languages. Psychon Bull Rev 10, 344–380 (2003). 12921412 10.3758/bf03196494 PMC3392189

[b40] Hamm J. P. & McMullen P. A. Effects of orientation on the identification of rotated objects depend on the level of identity. J Exp Psychol Hum Percept Perform 24, 413–426 (1998). 9606109 10.1037//0096-1523.24.2.413

[b41] Szekely A. & Bates E. Objective visual complexity as a variable in studies of picture naming. Center for Research in Language Newsletter 12, 3–33 www.crl.ucsd.edu/newsletter/12-2/article.html (2000). (Date of access: 1.9.2015.)

[b42] Derrington A. M., Krauskopf J. & Lennie P. Chromatic mechanisms in lateral geniculate nucleus of macaque. J Physiol 357, 241–265 (1984). 6512691 10.1113/jphysiol.1984.sp015499 PMC1193257

[b43] Stockman A. & Sharpe L. T. Spectral sensitivities of the middle- and long-wavelength sensitive cones derived from measurements in observers of known genotype. Vis Res 40, 1711–1737 (2000). 10814758 10.1016/s0042-6989(00)00021-3

[b44] Stockman A., Sharpe L. T. & Fach C. The spectral sensitivity of the human short-wavelength sensitive cones derived from thresholds and color matches. Vis Res 39, 2901–2927 (1999). 10492818 10.1016/s0042-6989(98)00225-9

[b45] Westland S., Ripamonti C. & Cheung V. Computational colour science using MatLab (second edition). Wiley. ISBN: 978-0-470-66569-5. (2012).

[b46] Wyszecki G. & Stiles W. S. Color science: concepts and methods, quantitative data and formulae (2nd edition ed.). New York: John Wiley & Sons. (2000).

[b47] Walsh J. W. T. Photometry (3rd edition). London, UK: Constable & Co. Ltd. (1958).

[b48] Ruppertsberg A., Wuerger S. M. & Bertamini M. The chromatic input of global motion perception. Visual Neurosci, 20, 421–428. (2003). 10.1017/s0952523803204077 14658770

[b49] Prins N. & Kingdom F. A. A. Palamedes: Matlab routines for analyzing psychophysical data. URL http://www.palamedestoolbox.org/ (2009).

[b50] Golz J. & MacLeod D. I. A. Colorimetry for CRT displays. JOSA A 20, 769–781 (2003). 12747426 10.1364/josaa.20.000769

